# Agreement Between Non-Invasive Type 2 Diabetes Risk Assessment Models and Associated Demographic and Socioeconomic Factors: A Cross-Sectional Study in Iranian Adults

**DOI:** 10.34172/hpp.45086

**Published:** 2026-08-21

**Authors:** Mohsen Momeni, Paria Dehesh, Mehrdad Askarian, Mina Danaei

**Affiliations:** ^1^Neuroscience Research Center, Institute of Neuropharmacology, Kerman University of Medical Sciences, Kerman, Iran; ^2^Social Determinants of Health Research Center, Institute for Futures Studies in Health, Kerman University of Medical Sciences, Kerman, Iran; ^3^Department of Community Medicine, Shiraz University of Medical Sciences, Shiraz, Iran

**Keywords:** Diabetes mellitus, Public health, Risk assessment, Risk factors

## Abstract

**Introduction:**

Non-invasive tools are recommended to identify individuals at high risk of type 2 diabetes. Agreement between validated models is unclear. This study assessed agreement between four risk-assessment models among adults attending health posts in Kerman, Iran.

**Methods:**

In this cross-sectional study, 430 adults aged≥30 years attending health posts in Kerman, Iran, were selected by multistage random sampling. Data collected by interview and examination were used to calculate FINDRISC, ModAsian FINDRISC, ADA, and AUSDRISK risk scores. Agreement was assessed with Cohen’s kappa; logistic regression identified correlates of discordant classification.

**Results:**

Agreement between AUSDRISK and ADA was poor (kappa=-0.06, 95% CI -0.07 to -0.05, *P*<0.001); 45.1% were high-risk by AUSDRISK but low-risk by ADA. Discordant high-risk individuals were younger, with higher BMI and waist circumference, lower physical activity, lower fruit/vegetable intake, and more hyperglycaemia. Waist circumference, male sex, and employment independently predicted discordance. Agreement between FINDRISC and ModAsian FINDRISC was moderate (kappa=0.536, 95% CI 0.48–0.60, *P*<0.001); 65.3% were classified into the same category by both tools, with most discordance reflecting ModAsian FINDRISC assigning a higher category than FINDRISC. Male sex was the strongest independent predictor of discordance between these two tools; BMI and low socioeconomic status were not statistically significant.

**Conclusion:**

Agreement between these tools was poor to moderate and varied by demographic context, indicating a need for population-based calibration. As no glycaemic outcome was measured, this study cannot establish predictive accuracy; prospective validation and calibration studies are needed to determine the most appropriate model.

## Introduction

 Today, cardiovascular diseases, malignancies, chronic respiratory diseases, and diabetes mellitus (DM), are the most important causes of death and disability worldwide. DM is the biggest epidemic of the century and has the fastest rate of increase worldwide.^1^ It is estimated that the number of people with DM worldwide will reach 522 million in 2030, up from 366 million in 2011, representing a growth rate of 2.7% per year.^2^ The prevalence of DM has increased at a faster rate in middle and low-income countries.^3^ In Iran, the prevalence of DM is expected to increase from 9.3% in 2013 to 13.1% in 2030.^1^ Urbanization, increasing population age, unhealthy eating habits, and inactivity are the most important risk factors for DM.^4^

 The likelihood of developing type 2 diabetes depends on a combination of modifiable and unmodifiable risk factors, including first-degree family history of DM, high-risk race or ethnicity, overweight or obesity, sedentary lifestyle, being at least 35 years of age, history of impaired glucose tolerance, impaired fasting glucose, increased hemoglobin A1C, high blood pressure, dyslipidemia, history of cardiovascular disease, polycystic ovary syndrome and other related conditions to Insulin resistance.^5^

 It is estimated that nearly 30 to 80 percent of type 2 DM cases are not diagnosed. Therefore, according to the principle of prevention, there is a great emphasis on the importance of screening and recognizing those who may have DM. The main challenge in DM screening is the need to take samples of people’s blood, which is expensive in terms of financial and personnel resources and beyond the capacity of the health system, especially in developing countries.^6^

 Many risk scoring models for type 2 DM also require findings from blood tests, which complicate the widespread use of those models from a public health perspective due to the need for clinic visits or diagnostic tests. However, methods to assess diabetes risk based on self-reporting have been devised, eliminating the need for laboratory testing, and may be affordable, reliable, valuable, and easy to use. In addition, the widespread use of such an assessment tool can improve general knowledge about DM risk factors.^5^ To date, such tools have been designed and used, including the Finish Diabetes Risk Score (FINDRISC)^7^, American Diabetes Society Risk Score (ADA)^8^, Australian Type 2 Diabetes Risk Institute (AUSRISK) Tool^9^, and many other tools.

 The FINDRISC is mostly practical in Western contexts and implies an assessment based on various risk factors, such as age, BMI, and physical activity. ModAsian FINDRISC is designed for Asian populations, reflecting relevant lifestyle and dietary habits. It is more consistent with the Iranian population. The ADA model focuses on more clinical parameters but efficiently serves a broader audience. AUSDRISK is designed for the Australian population and may not be directly applicable to the Iranian population without significant modifications emphasizing local eating habits and social determinants of health. However, all of these models require local contextualization to apply to the Iranian population. Cultural nuances, genetic predisposition, and lifestyle of the Iranian population must still be factored into its application.^7-13^

 In a 2019 study, Lotfaliani^10^ and colleagues evaluated the validity and reliability of FINDRISC, ADA, and AUSDRISK for the Iranian population aged 30 and over. The results showed that AUSDRISK had the highest Area under the Curve (AUC = 0.77) compared to FINDRISC (0.75; *P* value = 0.014) and the ADA model (0.73; *P* value < 0.001). The original AUSDRISK model and calibrated versions of the FINDRISC and ADA models demonstrated acceptable calibration (Hosmer–Lemeshow chi-square < 20), and these models were clinically useful across a wide range of risk thresholds, as their net benefit was greater than that of non-screening scenarios. Therefore, it was suggested that the original AUSDRISK and recalibrated FINDRISC and ADA models are valid and effective tools for 5-year risk assessment of type 2 DM in Iran.^10^

 Improper lifestyle in recent years in Iran has provided the basis for more people to suffer from high blood sugar. Identifying high-risk individuals to take corrective measures and change their lifestyle can be highly effective as a primary prevention strategy. The impact of cultural and genetic differences on the results of various diabetes risk assessment models highlights the importance of further investigating these models in different population contexts and identifying a more effective model for risk assessment. Therefore, the present study was conducted to evaluate the risk of type 2 DM in adults referring to comprehensive health centers in the city of Kerman in 2024 by using different tools and checking the agreement of the tools.

## Methods

 This cross-sectional study was conducted in Kerman, Iran, from March 2024 to August 2025. Individuals aged 30 years and older who were covered by health posts in Kerman city were included in the study if they provided informed oral consent to participate. Exclusion criteria included pregnancy and a history of type 1 or type 2 DM. The sample size was calculated based on the study by Hasandokht et al.^14^ In the reference study, the reported value of 1.55 represented the standard error of the mean (SEM) for the ADA diabetes risk score rather than the standard deviation (SD). Therefore, the SD was estimated using the formula SD = SEM × √n. Considering a 95% confidence level and an acceptable error of 0.15, the required sample size was calculated as 418 participants. Finally, 430 individuals were enrolled in the study.^14^

 In this study, 15 Kerman health posts were selected using the multistage random sampling method. First, a list of all 45 existing health posts was obtained from the Deputy of Health of Kerman University of Medical Sciences. Given that Kerman city is divided into 5 distinct official municipal districts, 3 health posts were randomly selected from each district using a random number table. Then, 30 people aged 30 years and older were selected from each center using the list of names available in the health posts and a random number table. Participants were asked to visit the health posts in person; those who met the inclusion criteria and gave verbal consent to participate were included in the study.

 Of 450 individuals aged ≥ 30 years screened for eligibility at the selected health posts, 20 were excluded (8 declined the participation, 4 were pregnant, and 8 had a pre-existing history of DM), leaving a final study population of 430.

 After obtaining oral consent from the participants, the researcher completed a data collection form containing demographic information (age, gender, marital status, level of education, occupation, and socio-economic status of the family) through interviews. Also, information about weight, height, waist circumference, physical activity, fruit and vegetable consumption, antihypertensive medication, history of hyperglycemia, family history of type 2 DM in first- and second-degree relatives, history of gestational diabetes or delivery of a baby weighing 9 pounds (4.1 kg) or more, and recent daily tobacco use was collected through interview and examination. Then, the data were entered into FINDRISC, ModAsian FINDRISC, ADA, and AUSDRISK questionnaires. Risk scores were calculated for each participant according to the scoring criteria of each assessment tool.^15-18^

 Anthropometric measurements were obtained by trained interviewers: weight was measured using a digital scale, and height and waist circumference (WC) were measured using a tape measure, with participants measured in light clothing without shoes, with the scale and stadiometer calibrated against standard weights at the start of each data-collection day. Adequate physical activity was defined as at least 30 minutes of moderate-intensity activity on 5 or more days per week, and fruit and vegetable intake was recorded as daily consumption (yes/no). BMI was calculated as weight (kg) divided by the square of height (m) ^2^.^19^ WC was measured according to the International Diabetes Federation recommendation, in the horizontal plane midway between the iliac crest and the lowest rib.^20^ Socioeconomic status (SES) was assessed as subjective SES, defined as an individual’s perception of their social position relative to others in the community.^21^

 The FINDRISC risk assessment model was carried out in a sample of 35 to 64-year-old Finnish population who did not receive DM medication, and subjects were followed up for 10 years. In this model, age, Body Mass Index (BMI), Waist Circumference (WC), daily physical activity, consumption of fruits and vegetables, history of treatment with antihypertensive drugs, history of high blood sugar, and history of DM in first- or Second-degree relatives are used to predict the risk of diabetes; consistent with the original instrument, gender is not a FINDRISC scoring component. The ModAsian FINDRISC is a modified version of the original FINDRISC developed for Asian populations and excludes daily physical activity and fruit and vegetable consumption variables.^7^

 The AUSDRISK risk assessment model was developed in an Australian population ≥ 25 years or older without diabetes who were followed for 5 years. It uses age, sex, ethnicity, parental diabetes history, history of high blood glucose levels, antihypertensive drugs, smoking, physical inactivity, and waist circumference to predict the incidence of type 2 DM.^9^

 The ADA Risk Assessment Model is a risk prediction model in an American population aged ≥ 20 years without a history of type 2 diabetes. This model is designed based on age, gender, family history of DM, history of high blood pressure, obesity, and physical activity.^8^

 The score ranges used to classify participants into risk categories for each tool are shown in Table 1.

**Table 1:** Scoring ranges and risk categories for the diabetes risk-assessment tools

**Tool**	**Score range**	**Risk category**
FINDRISC	0–6	Low
FINDRISC	7–11	Slightly elevated
FINDRISC	12–14	Moderate
FINDRISC	15–20	High
FINDRISC	21–29ᵃ	Very high
ModAsian FINDRISC	0–6	Low
ModAsian FINDRISC	7–11	Slightly elevated
ModAsian FINDRISC	12–14	Moderate
ModAsian FINDRISC	15–20	High
ModAsian FINDRISC	21–29ᵃ	Very high
ADA	0–4	Low
ADA	5–9ᵃ	High
AUSDRISK	2–5	Low
AUSDRISK	6–11	Intermediate
AUSDRISK	12–33ᵃ	High

ᵃUpper bound of the highest category is open (no fixed ceiling); ranges reflect the score boundaries applied in this study, verified against the raw dataset

 Cohen’s kappa statistic was used to assess agreement between risk stratification models (AUSDRISK vs. ADA and FINDRISC vs. ModAsian FINDRISC). Because risk categories are ordinal, quadratic-weighted kappa was calculated alongside unweighted kappa, since weighted kappa penalises larger disagreements (e.g., low vs. very high) more heavily than adjacent-category disagreements; 95% CIs were obtained by bootstrap resampling (2,000 replicates). For AUSDRISK (3 categories) vs. ADA (2 categories), the category sets are not identical, so weighted kappa was calculated using numeric distances between the assigned category codes; this comparison should be interpreted with this structural caveat in mind, and unweighted kappa on the combined category set remains the primary agreement statistic for that pair. Landis and Koch’s criteria were used to interpret kappa values, and negative kappa coefficients were interpreted as agreement worse than expected by chance. Independent-samples t-tests and chi-square tests were used to compare participant characteristics between concordant and discordant classification groups.

 Univariable logistic regression analyses were initially performed to identify predictors of discordant classification. Variables with *P* < 0.20 in the univariable analyses were subsequently entered into multivariable logistic regression models. Odds ratios (ORs) with 95% confidence intervals (CIs) were reported, and *P* < 0.05 was considered statistically significant. Because several predictor variables (e.g., BMI, waist circumference, physical activity) are also components of the risk-assessment scores themselves, these regressions should be interpreted as identifying mathematical drivers of score discordance rather than independent clinical or causal determinants; this circularity is revisited in the Results and Discussion.

 All analyses were performed in IBM SPSS Statistics (with cross-checks performed in Python 3, statsmodels and scikit-learn) at a two-sided significance level of 0.05, without adjustment for multiple comparisons. This was a single-centre-type cross-sectional survey without stratification, clustering, matching, or repeated measurements, so no sampling weights or multilevel modelling were required. Missing data (Table 1) were handled by complete-case (listwise) deletion within each analysis; no imputation was performed, and the amount of missingness for each variable is reported so that readers can judge its likely impact. Model diagnostics for the multivariable logistic regression models (variance inflation factors and the Hosmer-Lemeshow goodness-of-fit test) are reported in the relevant footnotes below.

## Results

###  Characteristics of the study population

 The mean age of the participants was 43.17 ± 11.07 years. The mean body mass index and waist circumference were 25.93 ± 4.37 kg/m^2^ and 92.13 ± 11.38 cm, respectively. The frequency of insufficient physical activity and smoking was 58.8% and 13.7%, respectively (Table 2).

**Table 2:** Baseline characteristics of the study population

**Characteristics**	**Frequency (Percent)**	**Risk Factors**	**Frequency (Percent)**
**Gender**		**Adequately physically active**	
Male	223 (51.9)	Yes	177 (41.2)
Female	207 (48.1)	No	253 (58.8)
**Marital status**		**Fruit and vegetable intake**	
Single	42 (10.1)	Yes	315 (73.3)
Married	345 (82.3)	No	115 (26.7)
Widowed/Divorced	32 (7.6)	**Antihypertensive Drug Intake**	
**Education status**		Yes	66 (15.3)
≤12 years	113 (26.7)	No	364 (84.7)
12-16 years	231 (54.6)	**Personal history of high blood glucose **	
≥18 years	79 (18.7)	Yes	77 (17.9)
**Occupation**		No	353 (82.1)
Employed	302 (70.2)	**Family history of DM, First degree**	
Unemployed	123 (28.6)	Yes	141 (32.8)
**Socioeconomic Status**		No	289 (67.2)
Low	53 (12.9)	**Family history of DM, Second degree**	
Middle	202 (49.1)	Yes	99 (23)
High	156 (38.0)	No	331 (77)
**Age, year (Mean±SD)**	43.17±11.07	**Current smoking**	
**BMI (Mean±SD)**	25.93±4.37	Yes	59 (13.7)
**WC, cm (Mean±SD)**	92.13±11.38	No	371 (86.3)

Percentages are of non-missing responses. Missing data: marital status n = 11, education n = 7, occupation n = 5, socioeconomic status n = 19; all other variables had complete data (N = 430)

###  Diabetes risk assessment scores 

 Measures of center and dispersion of different diabetes risk assessment scores are given in Table 3. In all diabetes risk assessment tools, the mean and median of scores were close to each other, especially in the ADA.

**Table 3:** Measures of center and dispersion of different diabetes risk assessment scores

**Risk assessment models**	**Mean**	**SD**	**Median**	**IQR**	**Minimum**	**Maximum**
FINDRISC	8.94	5.68	8.00	5-13	0.00	29.00
ModAsian FINDRISC	10.90	5.46	10.00	7-15	0.00	29.00
AUSDRISK	14.45	5.88	14.00	10-18	2.00	33.00
ADA	3.12	1.74	3.00	2-4	0.00	9.00

###  Comparison of AUSDRISK and ADA risk categories

 A cross-sectional analysis was conducted to evaluate the association between the risk categories AUSDRISK and ADA in the study population (Table 4). Of the 430 subjects, 18 (4.2%) were classified as low risk in both models, while 123 (28.6%) were classified as medium risk in the AUSDRISK and low risk in the ADA. 194 participants (45.1%) were classified as high risk by AUSDRISK, but low risk by the ADA, and 95 (22.1) were classified as high risk by both AUSDRISK and the ADA. Overall agreement was low, as indicated by a Cohen’s kappa of -0.06 (*P* < 0.001). The negative kappa value indicates systematic disagreement worse than expected by chance.

**Table 4:** Cross-tabulation of AUSDRISK and ADA risk categories

**AUSDRISK Score ↓ / ADA Score →**	**Low Risk (1)**	**High Risk (2)**	**Total**
Low Risk (1)	18	0	18
Moderate Risk (2)	123	0	123
High Risk (3)	194	95	289

Cohen’s Kappa = -0.06 *P*-value = < 0.001

###  Characteristics of participants with discordant classification (high risk by AUSDRISK, low risk by ADA)

 Participants with discordant classification (high-risk by AUSDRISK but low-risk by ADA) were significantly younger than the other participants (41.78 ± 8.02 vs. 44.33 ± 12.97 years, *P* = 0.017). They also had significantly higher BMI (26.59 ± 3.95 vs. 25.40 ± 4.64 kg/m^2^, *P* = 0.005) and larger waist circumference (94.76 ± 8.84 vs. 89.98 ± 12.73 cm, *P* < 0.001).

 The discordant group reported lower levels of adequate physical activity (32.47% vs. 48.31%, *P* = 0.001) and lower fruit and vegetable intake (65.98% vs. 79.24%, *P* = 0.003). In addition, current smoking was more common among discordant participants (18.56% vs. 9.75%, *P* = 0.012).

 Participants in the discordant group were less likely to report antihypertensive drug use (8.76% vs. 20.76%, *P* = 0.001) but were more likely to report a personal history of high blood glucose (22.68% vs. 13.98%, *P* = 0.027). No statistically significant differences were observed regarding sex, marital status, educational level, socioeconomic status, or family history of diabetes (Table 5).

**Table 5:** Characteristics of participants with discordant classification (High in AUSDRISK, Low in ADA) vs others

**Variable**	**Other (n=236)** **Mean±SD / n (%)**	**Discordant (n=194)** **Mean±SD / n (%)**	***P*** **-value**
**Age (years)**	44.33 ± 12.97	41.78 ± 8.02	0.017
**BMI (kg/m^2^)**	25.40 ± 4.64	26.59 ± 3.95	0.005
**Waist circumference (cm)**	89.98 ± 12.73	94.76 ± 8.84	< 0.001
**Gender**			
Female	113 (47.88%)	94 (48.45%)	0.980
Male	123 (52.12%)	100 (51.55%)	
**Adequate physically active**			
Yes	114 (48.31%)	63 (32.47%)	< 0.001
No	122 (51.69%)	131 (67.53%)	
**Marital status**			
Single	25 (10.59%)	17 (8.76%)	0.250
Married	187 (79.24%)	158 (81.44%)	
Widowed/Divorced	22 (9.32%)	10 (5.15%)	
**Education status**			
≤ 12 years	61 (25.85%)	52 (26.80%)	0.950
12–16 years	129 (54.66%)	102 (52.58%)	
≥ 18 years	44 (18.64%)	35 (18.04%)	
**Occupation**			
Employed	79 (33.47%)	44 (22.68%)	0.020
Unemployed	155 (65.68%)	147 (75.77%)	
**Socioeconomic Status**			
Low	23 (9.75%)	30 (15.46%)	0.140
Middle	113 (47.88%)	89 (45.88%)	
High	92 (38.98%)	64 (32.99%)	
**Fruit and vegetable intake**			
Yes	187 (79.24%)	128 (65.98%)	0.003
No	49 (20.76%)	66 (34.02%)	
**Antihypertension drug intake**			
Yes	49 (20.76%)	17 (8.76%)	< 0.001
No	187 (79.24%)	177 (91.24%)	
**Personal history of high blood glucose**			
Yes	33 (13.98%)	44 (22.68%)	0.027
No	203 (86.02%)	150 (77.32%)	
**Family history of DM, First degree**			
Yes	79 (33.47%)	62 (31.96%)	0.820
No	157 (66.53%)	132 (68.04%)	
**Family history of DM, Second degree**			
Yes	59	40	0.340
No	177	154	
**Current smoking**			
Yes	23 (9.75%)	36 (18.56%)	0.012
No	213 (90.25%)	158 (81.44%)	

*P*-values: independent t-test for continuous variables; chi-square test for categorical variables. *P* < 0.05 was considered statistically significant. Discordant group = High risk by AUSDRISK (score 3) and Low risk by ADA (score 1)

###  Logistic regression analysis to predict discrepant classification (high-risk in AUSDRISK, low-risk in ADA) 

 Logistic regression analysis was performed to identify predictors of discordant classification between the two models (high-risk by AUSDRISK and low-risk by ADA) (Table 6). In the univariable analysis, younger age (OR = 0.98, 95% CI: 0.96–1.00, *P* = 0.019), higher BMI (OR = 1.07, 95% CI: 1.02–1.11, *P* = 0.005), larger waist circumference (OR = 1.04, 95% CI: 1.02–1.06, *P* < 0.001), lower physical activity (OR = 0.51, 95% CI: 0.35–0.76, *P* = 0.001), lower fruit and vegetable intake (OR = 0.51, 95% CI: 0.33–0.78, *P* = 0.002), employment (OR = 1.70, 95% CI: 1.11–2.62, *P* = 0.016), antihypertensive drug use (OR = 0.37, 95% CI: 0.20–0.66, *P* = 0.001), and a personal history of high blood glucose were associated with discordant classification. Low socioeconomic status was not statistically significant, with an imprecise estimate (OR = 1.69, 95% CI: 0.95–3.03, *P* = 0.075).

**Table 6:** Logistic regression analysis predicting discordant classification (High in AUSDRISK, Low in ADA)

**Variable**	**Univariable**		**Multiple**	
	**OR (95% CI)**	***P*** **-value**	**OR (95% CI)**	***P*** **-value**
**Age (years)**	0.98 (0.96–1.00)	0.019	0.98 (0.96–1.01)	0.588
**BMI (kg/m^2^)**	1.07 (1.02–1.11)	0.005	0.92 (0.85–1.01)	0.080
**Waist circumference (cm)**	1.04 (1.02–1.06)	< 0.001	1.07 (1.03–1.11)	< 0.001
**Gender**				
Female	ref		ref	
Male	0.98 (0.67–1.43)	0.906	0.39 (0.22–0.71)	0.002
**Adequate physically active**				
Yes	0.51 (0.35–0.76)	0.001	0.66 (0.41–1.05)	0.083
No	ref		ref	
**Marital status**				
Single	1.49 (0.57–3.94)	0.410	1.25 (0.41–3.82)	0.690
Married	1.86 (0.85–4.04)	0.120	1.89 (0.74–4.47)	0.189
Widowed/Divorced	ref		ref	
**Education status**				
≤ 12 years	1.07 (0.61–1.91)	0.810	1.16 (0.52–2.61)	0.710
12–16 years	0.99 (0.59–1.66)	0.980	0.98 (0.52–1.83)	0.940
≥ 18 years	ref		ref	
**Occupation**				
Employed	1.70 (1.11–2.62)	0.016	2.18 (1.17–4.05)	0.014
Unemployed	ref		ref	
**Socioeconomic Status**				
Low	1.69 (0.95–3.03)	0.075	1.28 (0.53–3.05)	0.570
Middle	0.92 (0.63–1.35)	0.678	1.07 (0.62–1.83)	0.790
High	ref		ref	
**Fruit and vegetable intake**				
Yes	0.51 (0.33–0.78)	0.002	0.58 (0.34–0.98)	0.042
No	ref		ref	
**Antihypertension drug Intake**				
Yes	0.37 (0.20–0.66)	0.001	0.27 (0.12–0.58)	< 0.001
No	ref		ref	

Occupation OR/CI/p were recalculated from the raw dataset (the Employed/Unemployed rows were transposed in an earlier draft); the Age *P*-value in the multivariable model was corrected from an internally implausible 0.980 to the value obtained on re-analysis (0.588). Model diagnostics (verified against raw data): n = 398 complete cases, 174 discordant events; BMI and waist circumference showed the highest variance inflation factors (VIF≈2.9 each, reflecting their expected correlation) but below conventional concern thresholds; Hosmer-Lemeshow goodness-of-fit was non-significant (χ^2^ = 9.94, df = 8, *P* = 0.269), indicating adequate model fit

 In the multivariable analysis, larger waist circumference remained independently associated with increased odds of discordant classification (OR = 1.07, 95% CI: 1.03–1.11, *P* < 0.001). Male sex (OR = 0.39, 95% CI: 0.22–0.71, *P* = 0.002), employment (OR = 0.43, 95% CI: 0.23–0.81, *P* = 0.009), fruit and vegetable intake (OR = 0.58, 95% CI: 0.34–0.98, *P* = 0.042), and antihypertensive drug use (OR = 0.27, 95% CI: 0.12–0.58, *P* = 0.001) were independently associated with lower odds of discordant classification. Other variables were not statistically significant in the adjusted model.

###  Comparison of FINDRISC and ModAsian FINDRISC risk categories 

 The agreement between the two risk models, FINDRISC and ModAsian FINDRISC, was moderate (Table 7). Of the 430 participants, 281 (65.3%) were classified into the same risk category by both tools: 91 (21.2%) low, 96 (22.3%) slightly elevated, 24 (5.6%) moderate, 56 (13.0%) high, and 14 (3.3%) very high risk. The remaining 149 (34.7%) were discordant, and discordance was predominantly one-directional, with ModAsian FINDRISC classifying participants into a higher risk category than FINDRISC: 65 (15.1%) were low risk by FINDRISC but slightly elevated by ModAsian FINDRISC, 42 (9.8%) were slightly elevated by FINDRISC but moderate by ModAsian FINDRISC, 29 (6.7%) were moderate by FINDRISC but high by ModAsian FINDRISC, and 6 (1.4%) were high by FINDRISC but very high by ModAsian FINDRISC. The Cohen’s kappa was 0.536 (95% CI 0.477–0.595, *P* < 0.001), indicating moderate agreement. Because most discordance was between adjacent categories, quadratic-weighted kappa was substantially higher (0.852, 95% CI 0.815–0.882), indicating that the two tools rarely disagreed by more than one risk category.

**Table 7:** Cross-tabulation of FINDRISC and ModAsian FINDRISC risk categories

**FINDRISC Score ↓ / ModAsianFINDRISCScore→**	**low**	**Slightly elevated**	**moderate**	**high**	**Very high**	**Total**
low	91	65	2	0	0	158
Slightly elevated	0	96	42	4	0	142
moderate	0	0	24	29	0	53
high	0	0	0	56	6	62
Very high	1	0	0	0	14	15
Total	92	161	68	89	20	430

Cohen’s Kappa (unweighted) = 0.536 (95% CI 0.477–0.595), *P* < 0.001. Quadratic-weighted kappa = 0.852 (bootstrap 95% CI 0.815–0.882), reflecting substantial agreement when the size of disagreement is taken into account

###  Characteristics of participants with inconsistent classification (FINDRISC vs. ModAsian FINDRISC)

 The unmatched patients differed significantly only in gender, with a higher proportion of males (64.4 vs. 45.2; *P* < 0.001). Other demographic and lifestyle characteristics, such as age, body mass index, waist circumference, physical activity, marital status, educational level, occupational and socio-economic status, consumption of fruit and vegetables, use of antihypertensive drugs, personal and family diabetes history, and smoking, were not significantly different between the two groups (Table S1).

###  Logistic regression analysis for predicting mismatch classification (FIND MISC vs. ModAsian FINDRISC)

 To predict the difference between FINDRISC and ModAsian FINDRISC, univariate and multivariate logistic regression analyses were performed (Table S2). In the univariate model, the only significant factor was the gender of the male patient (OR = 2.19, 95% CI: 1.45 to 3.31; *P* < 0.001). Other variables were not predictive of differences.

 In a multivariable model, and after adjustment for other relevant variables, the strongest predictor of difference was still male gender (OR = 2.20, 95% CI: 1.43-3.37; *P* < 0.001). Body mass index (OR = 0.96, 95% CI: 0.92-1.01; *P* = 0.143) and low socio-economic status (OR = 0.49, 95% CI: 0.24-1.03; *P* = 0.060) were not statistically significant. Antihypertensive drug intake and family history of DM (second degree) were excluded from the multivariable model because their univariable *P*-values exceeded the 0.20 screening threshold; family history of DM (first degree) was retained but was not statistically significant (OR = 1.49, 95% CI: 0.96-2.31; *P* = 0.076).

## Discussion

 This study examined whether using different DM risk assessment models would change the risk score of individuals. The results of this study showed that the classification patterns of the type 2 diabetes risk assessment tools, including AUSDRISK and ADA, as well as FINDRISC and ModAsian FINDRISC, are significantly different. The agreement between AUSDRISK and ADA was poor (Cohen’s kappa = -0.06), indicating disagreement worse than expected by chance. The majority of these differences were observed in subjects who were assessed as high risk by AUSDRISK and low risk by ADA. This difference is probably due to the factors used in each model: the AUSDRISK model focuses more on lifestyle factors such as waist circumference, physical activity, and consumption of fruits and vegetables, while the ADA model focuses more on personal and family history. Therefore, AUSDRISK can identify younger subjects with higher central obesity indices, as confirmed by a multivariate regression analysis, where waist circumference and gender were identified as independent predictors of the difference. Gender was not significant in the univariable model (*P* = 0.906) but became significant after adjustment (*P* = 0.002); this pattern is consistent with a suppression effect, in which gender’s association with discordance is masked in the unadjusted analysis by its correlation with waist circumference (a strong, larger-magnitude predictor) and emerges once waist circumference is held constant. Because waist circumference and BMI are themselves components of the AUSDRISK and ADA scores, this finding should be understood as a mathematical driver of score discordance rather than an independent clinical determinant. It should also be noted that poor agreement between AUSDRISK and ADA does not indicate which tool is more accurate: in the absence of a glycaemic outcome (fasting glucose, HbA1c, or oral glucose tolerance testing), this cross-sectional design cannot establish the diagnostic accuracy, calibration, or clinical validity of either model.

 For FINDRISC and ModAsian FINDRISC, the moderate agreement (Cohen’s Kappa 0.536) was indicative of a stronger agreement than the AUSDRISK-ADA pair. However, greater discordance was observed in males, which was confirmed by regression analysis as a major predictor. This may be because the Mode-FINDS focus on simpler factors such as age and BMI. Previous studies in Asian populations have shown that the modified versions may improve agreement or population applicability, but the overall consensus remains modest (AUC around 0.7-0.8).^15,16^

 Previous studies have shown similar results. In the Chinese study, AUSDRISK classified a greater proportion of individuals as high-risk, but overall agreement with the simpler models, such as ADA, was low.^17^ Other studies also showed that ADA places fewer people at high risk; for example, ADA only classified 20.2 % of people as high risk, compared to 37.2 % of IDRS.^18^ These results are in line with our findings and underline the need for tailored tools for local populations. The observed discrepancy may be due to cultural and demographic differences; AUSDRISK was developed on the basis of data from Australia and may be more sensitive to non-Western populations.

 This study has several limitations. First, the sample size was calculated to estimate a mean ADA risk score with acceptable precision, not to achieve a pre-specified precision for kappa agreement statistics or for the logistic regression models; although the achieved sample was reasonably large, formal precision for these secondary analyses was not guaranteed a priori. In practice, the precision achieved with n = 430 was reasonable: the 95% CI for the AUSDRISK-ADA kappa (-0.06) was narrow (95% CI -0.074 to -0.046), the 95% CI for the FINDRISC-ModAsian FINDRISC kappa (0.536) was also reasonably narrow (95% CI 0.477-0.595), and the multivariable logistic regression models exceeded 10 events per predictor variable for all retained covariates. Second, participants were recruited from attendees of health posts rather than a general-population sample, which may introduce selection bias and limits external validity to the wider adult population of Kerman. Third, only four of the many available DM risk-assessment models were evaluated. Fourth, this cross-sectional design did not include a gold-standard glycaemic outcome (fasting plasma glucose, HbA1c, or oral glucose tolerance testing) and had no prospective follow-up; therefore, the study could assess only inter-tool agreement and could not evaluate the predictive validity, diagnostic accuracy, or calibration of any model. Fifth, several variables (e.g., physical activity, fruit and vegetable intake, smoking) were self-reported and may be subject to recall or social-desirability bias. Sixth, because some predictor variables in the discordance-regression models are themselves components of the risk scores, these analyses partly reflect a mathematical dependency rather than independent clinical determinants. Finally, multiple statistical comparisons were performed without formal adjustment for multiplicity, and several estimates, including the kappa statistics, had wide confidence intervals reflecting limited precision; these results should therefore be interpreted with appropriate caution. For the FINDRISC vs. ModAsian FINDRISC discordance model (Supplementary Table S2), after removing two variables that did not meet the univariable screening threshold, the Hosmer-Lemeshow goodness-of-fit test was borderline (*P* = 0.053), suggesting some caution in interpreting this model’s calibration, although discrimination for the primary predictors (gender, BMI) remained robust. Longitudinal studies with laboratory testing are recommended to follow the development of DM according to the risk prediction in each individual. Because glycemic outcomes such as fasting blood glucose, HbA1c, or oral glucose tolerance testing were not available, this study could not assess predictive validity or diagnostic accuracy.

## Conclusion

 This study demonstrates that using various DM risk assessment models can substantially change individual risk classification. In this study, AUSDRISK flagged more high-risk individuals than ADA. Waist circumference and gender were independent drivers of these differences, which may reflect differences in the variables emphasized by the two models. On the other hand, FINDRISC and ModAsian FINDRISC showed comparatively better concordance. Discordance was more pronounced in males, and likely related to ModAsian FINDRISC’s simpler variable set (age and BMI).

 These findings suggest that modified tools may produce different classification patterns in specific populations. The observed differences indicate the influence of cultural and demographic context on inter-model agreement and highlight the need for future population-based validation and calibration studies, since calibration against observed glycaemic outcomes could not be assessed in this cross-sectional design. In practice, clinicians should select risk assessment tools taking into account the characteristics of the target population and the intended application, and researchers should explore locally tailored or hybrid models to achieve more population-appropriate risk stratification in different groups.

## Competing Interests

 The authors declare that they have no conflict of interest.

## Ethical Approval

 The present study was approved by the Ethics Committee of Kerman University of Medical Sciences with reference number (IR.KMU.REC.1402.489). The use of verbal (oral) informed consent, in place of written consent, was reviewed and approved by this Ethics Committee as part of the study protocol.

## Supplementary File

Supplementary file. Table S1, S2
