# Supplementary material for: Agreement Between Non-Invasive Type 2 Diabetes Risk Assessment Models and Associated Demographic and Socioeconomic Factors: A Cross-Sectional Study in Iranian Adults
Source: Health Promot Perspect. 2026 Aug 21;16(2):209–16. doi: 10.34172/hpp.45086 (PMC13723382; doi:10.34172/hpp.45086)
Supplement: Supplementary file 1 [file hpp-16-209-s001.pdf]

Momeni et al, Health Promotion Perspectives 2026;16(2):209-S1
doi:10.34172/hpp.45086
https://hpp.tbzmed.ac.ir

Characteristics of participants with discordant classification (FINDRISC vs. ModAsian FINDRISC).

Discordant = participants classified into different risk categories by FINDRISC and ModAsian FINDRISC;

Other = all remaining participants classified into the same category by both tools.

Table S1. Characteristics of participants with discordant classification (different FINDRISC vs ModAsian FINDRISC)

Variable	Other (n = 281) Mean ± SD / n (%)	Discordant (n = 149) Mean ± SD / n (%)	p-value
**Age (years)**	42.84±10.82	43.80±11.54	0.390
**BMI (kg/m²)**	26.15±5.11	25.53±2.41	0.160
**Waist circumference (cm)**	92.34±12.71	91.74±8.18	0.610
**Gender**			
Female	154(54.8)	53(35.57)	<0.001
Male	127(45.2)	96(64.43)	
**Adequate physically active**			
Yes	117(41.63)	60(40.26)	0.830
No	164(58.36)	98(65.77)	
**Marital status**			
Single	32(11.38)	10(6.71)	0.340
Married	224(79.71)	121(81.21)	
Widowed/Divorced	20(7.11)	12(8.05)	
**Education status**			
≤12 years	79(28.11)	34(22.81)	0.410
12-16 years	146(51.95)	85(57.04)	
≥18 years	54(19.21)	25(16.77)	
**Occupation**			
Employed	83(29.53)	40(26.84)	0.650
Unemployed	196(69.75)	106(71.14)	
**Socioeconomic Status**			
Low	40(14.23)	13(8.72)	0.170
Middle	125(44.48)	77(51.67)	
High	102(36.29)	54(36.24)	
**Fruit and vegetable intake**			
Yes	202(71.88)	113(75.83)	0.430
No	79(28.11)	36(24.16)	
**Antihypertension drug Intake**			

Yes	39(13.87)	27(18.12)	0.260
No	242(89.12)	122(81.87)	
**Personal history of high blood glucose**			
Yes	52(18.51)	25(16.77)	0.690
No	229(81.49)	124(83.22)	
**Family history of DM, First degree**			
Yes	85(30.24)	56(37.58)	0.132
No	196(69.75)	93(62.41)	
**Family history of DM, Second degree**			
Yes	60(21.35)	39(26.17)	0.270
No	221(78.64)	110(73.82)	
**Current smoking**			
Yes	43(15.30)	26(17.44)	0.580
No	238(84.69)	123(82.55)	

Logistic regression analysis for predicting discordant classification (FINDRISC vs. ModAsian FINDRISC). Outcome = 1 if a participant's FINDRISC and ModAsian FINDRISC risk categories differed (discordant), 0 if they matched. Because several predictors are themselves components of the risk scores, associations below should be interpreted as correlates of score discordance rather than independent causal determinants.

Table S2. Logistic regression analysis predicting discordant classification between FINDRISC and ModAsian FINDRISC

Variable	Univariable			Multiple		
	OR	95% CI	p-value	OR	95% CI	p-value
**Age (years)**	1.01	0.99-1.02	0.390			
**BMI (kg/m²)**	0.96	0.92-1.02	0.160	0.96	0.92-1.01	0.143
**Waist circumference (cm)**	0.99	0.97-1.01	0.610			
**Gender**						
Female	ref			ref		
Male	2.19	1.45-3.31	<0.001	2.20	1.43-3.37	<0.001
**Adequate physically active**						
Yes	0.94	0.63-1.41	0.780			
No	ref					
**Marital status**						
Single	0.51	0.19-1.42	0.210			

Married	0.90	0.42-1.90	0.780			
Widowed/Divorced	ref					
**Education status**						
≤12 years	0.93	0.49-1.73	0.810			
12-16 years	1.26	0.73-2.16	0.410			
≥18 years	ref					
**Occupation**						
Employed	1.07	0.69-1.65	0.764			
Unemployed	ref					
**Socioeconomic Status**						
Low	0.61	0.31-1.24	0.170	0.49	0.24-1.03	0.060
Middle	1.16	0.75-1.79	0.490	1.11	0.71-1.74	0.652
High	ref			ref		
**Fruit and vegetable intake**						
Yes	1.22	0.77-1.93	0.370			
No	ref					
**Antihypertension drug Intake**						
Yes	1.37	0.81-2.34	0.240			
No	ref					
**Personal history of high blood glucose**						
Yes	1.12	0.66-1.90	0.650			
No	ref					
**Family history of DM, First degree**						
Yes	1.38	0.91-2.10	0.120	1.49	0.96-2.31	0.076
No	ref			ref		
**Family history of DM, Second degree**						
Yes	1.31	0.82-2.07	0.250			
No	ref					
**Current smoking**						
Yes	1.17	0.68-1.99	0.560			
No	ref					
